# Current Status of Long-term Care in Taiwan: Transition of Long-term Care Plan From 1.0 to 2.0

**DOI:** 10.15171/ijhpm.2019.115

**Published:** 2019-11-30

**Authors:** Chih-Ching Yang, Jui-Yuan Hsueh, Cheng-Yu Wei

**Affiliations:** Taiwan Ministry of Health and Welfare, Taipei City, Taiwan.

## Dear Editor,

Population aging is an issue that all developed countries in the world are facing today. In 1993, Taiwan officially entered the aging society and fertility rate has been less than 2 since 1985, and trending less than 1.5 in the past decade. The situation of having fewer children was getting severe, so that our number of members per household has dropped from 3.57 to 2.70 in the past 20 years. By 2018, Taiwan has became an aged society. Therefore, Taiwan government needed to prepare the responses to the challenges brought out by the rapid population aging. At first, let us take an overview of the development of long-term care (LTC) policy in Taiwan. Since 2007, the Central Government has been establishing LTC service policies^1^. Taiwan government launched the LTC 10-year plan version 1 (LTC 1.0)^2^ in 2007. The Long-term Care Services Act was passed-by in June of 2015. This Act provides the legislative base of constructing LTC services system. Although the LTC 1.0 has performed well, Taiwan government found that adjustments were needed to fit the needs of the public better, therefore, in 2016, passed the LTC plan version 2.0 (LTC 2.0). As to the current status of the LTC Insurance Law Draft^3,4^: the draft was sent into Legislative Yuan twice, but it was withdrawn after President Tsai took office. The bill was tackled mainly due to the issue of worker shortage and inadequate supply of services.

In order to build a continuum of care which is person-centered and community-based, Taiwan government planned to gradually establish an accessible, affordable, good-quality and universal service system that could provide diversified continuous services, including family support services, home and community-based, and institutional-based programs. Furthermore, it is a system that upstream prevention to delay disability,^5,6^ and downstream preparedness to provide discharge plan and home-based medical care. This system will accomplish the goal of “aging in place” for older adults and give them the opportunity to age in a community with disability and give them a place they are familiar with. As the other countries, in Taiwan, the eligibility criteria for public funded LTC services are set on functional limitations and age. In LTC 1.0, the service target majorly focused on older people with disability. Because some special group members experience aging process at younger age, such as indigenous people with disability, therefore Taiwan government lowered the age of care recipients to 55 y/o in LTC 2.0. In addition, Taiwan government considered the needs of special groups and incorporated all the possible population with service need, such as people with dementia aged over than 50, plain-land indigenous people with functional limitations (aged 55 to 64), people with disability (aged under 49), older people with frailty (aged 65 and over). With the expansion of service target, the number of population in need for LTC services increased above 40% until now. In this “person-centered” LTC service system, care managers serve as the gate-keeper for publically funded care/support. The care managers evaluated the case with need assessments and based on the assessment results, the benefit level was determined and appropriate care program was approved, then linked to the relevant service resources. Once the services have been delivered, the care manager should conduct regular reviews and monitors to ensure service quality. Besides, in LTC 1.0, most, but not all, providers delivered one single service which could not meet people’s diverse needs, so Taiwan government modified in LTC 2.0 to provide more diverse, flexible and integrated services to meet people’s needs. The original need assessment tool (ADL scale) is not inclusive enough, and failed to include cognitive impairment, and special care need, therefore, the needs assessment tool was revised, and the needs of LTC were divided into 8 levels with evidence-based data, to reflecting various physical, mental disability and dementia. As for the payment for LTC providers is fee for service, Taiwan government modified the benefit system to bundle payment, to provide user-centered integrated services. In accordance with the Government Procurement Law, the LTC service is commissioned to providers by public sector. The write-off procedure is too complex and time-consuming, therefore the collaboration between public and private sector was changed from “commissioned” to “contracted.”

In LTC 2.0, the service items was expanded from 8 to 17 services items, including dementia care service, small-scale multi-functional services, family support services, one innovative integrated service system, two programs of prevention or delay of disability, and two connection services on discharge plan from hospital to home-based medical care. Meanwhile, Taiwan government have constructed a comprehensive community care service system with A, B, and C tiers with the goal to integrate medical care,^7^ LTC services, housing, prevention, and assistance to allow people with disability to receive the care they need within a 30-minute drive. Apart from all of the above, LTC resources were constructed with the concept “aging in place” with special budget of forward-looking infrastructure (US$0.247 billion). Till September 2019, Taiwan government has already established 580A-43957B-2435C for more than 230 000 LTC demanders, but there is still lack of about 50 000 beds in institutional LTC centers. Those beds will be intended to be established within four years with planned US$0.17 billion expenditure.

The challenges Taiwan government still face in the future are the source of funding for implementing LTC 2.0 mainly from taxation and regulatory instrument to assure the quality. For the quality improvement, information technology-based techniques such as wearable device with artificial intelligence may be needed to be adopted. Finally, a smart community-based system to provide quality, affordable and popular services through this second 10-year plan for LTC will be anticipated in Taiwan.

## Ethical issues

Not applicable.

## Competing interests

Authors declare that they have no competing interests.

## Disclaimer

The opinions expressed in this article are the authors’ and do not necessarily reflect those of their agency.

## Authors’ contributions

JYH: Conception and design, administrative, technical, or material support, and Supervision; CYW: data collection, data analysis and interpretation, drafting of the manuscript; CCY: Conception and design, drafting of the manuscript, data interpretation, administrative, technical, or material support, supervision, critical review.
